# Justice Before Pluriversality—A Response to Jecker et al

**DOI:** 10.1007/s11673-025-10444-5

**Published:** 2025-03-14

**Authors:** Zohar Lederman

**Affiliations:** Department of Emergency Department, University of Hong Kong, 5 Sassoon Rd, Pok Fu Lam, Hong Kong

**Keywords:** Pluriversality, Palestinians, Israel

## Introduction

In this commentary, I argue that the way in which pluriversality is exemplified by Jecker et al. (2025) is self-defeating. While my argument, if compelling, does not entail that pluriversality as an ideal is wrong, it at the very least makes it much more suspicious. If the case for pluriversality is to succeed, it will require a much more careful analysis that is sensitive to historical, social, and political contexts. The main reason this present attempt to justify pluriversality fails is that the examples used by Jecker et al. are instances of a violation of the very condition posed by the authors to be necessary for the legitimization of pluriversality: the avoidance of destroying other worlds.

## The Case for Pluriversalism

My friends, colleagues, and mentors argue for the re-introduction of pluralism to bioethics, pluralism that includes not only differing religious worldviews but also existences and ontologies i.e. pluriversality. Such pluriversalism, say Jecker et. al. (2025) is warranted as long as the different worlds “… avoid harming people or destroying other worlds.” In a footnote, Jecker et al. explain that destroying other worlds means “eliminating other ways of knowing, being, and acting.” Jecker et al. are careful to emphasize that their approach is not synonymous with moral relativism. Rather, when different worlds do destroy other worlds, we should be able to reproach others who do not share our world: “Pluriversality challenges each of us to be ethical, not just within our own world but within a world of many worlds.”

Jecker et al. posit five ethical restraints necessary to enable pluriversalism. One of these restraints is justice, understood in part as “each individual who wishes to contribute to bioethics has a fair chance to give testimony and have it received as prima facie credible.” Justice also includes a subjective component, meaning that contributors to bioethics feel they are being given a fair chance to give reliable testimony. Another restraint is tolerance. A single unifying definition of tolerance is not readily offered by Jecker et al., but for present purposes one may understand it in two ways. First, toleration “requires avoiding judging others with undue severity.” Second, tolerance means epistemic and ethical humility, or the ability to shape and revise worldviews based on evidence or arguments rather than surrender to dogma.

Jecker et al. then make a case to apply a pluriversality approach to religious contributions to bioethics, praising the *Journal of Bioethical Inquiry* for “openness to religious contributions.” The term “religious contributions” is in fact mentioned eleven times by the authors, but it is never explained. An analysis of three examples mentioned by them raises the suspicion that the authors themselves are unclear as to what this term means.

## What is “Religious” About “Religious Contributions”?

These three examples are: (Lederman and Halberthal 2022; Komesaroff 2024; Rashi 2024). The problem is that these publications all take a very different approach in making their case. Lederman and Halberthal explicitly make a humble argument grounded in reason that is merely informed by key Islamic principles and methods. Their reliance on Islamic texts has two goals: a philosophical, academic goal, to enrich their reasonable argument, and a pragmatic goal, to speak to Muslim Healthcare professionals in a language that they might find most appealing. If an interlocutor were to question the interpretation of the Islamic principles and methods, Lederman and Halberthal may concede that their interpretation was indeed wrong but still hold on to their overall argument.

In the case of Komesaroff, the only explicit religious reference is to the Jewish scholar Levinas. If an interlocutor were to question Judaism or even Levinas for that matter, Komesaroff’s argument would still hold.

In complete contrast to these two publications, Rashi (2024, 58) explicitly and wholeheartedly relies on the Jewish religion and tradition to ground his argument:

One must take as a starting point and understand the meaning of the religious duty to take care of the other’s medical needs in general and the proper ethical treatment of prisoners in particular. In both areas it is not the individual’s right that is in question but a religious and humane obligation. (Rashi 2024, 58)

If an interlocutor were to effectively question Rashi’s interpretation of Judaic texts and compellingly argue instead that according to Judaism prisoners should not be vaccinated, then Rashi would have no choice but to concede and modify his main argument. Why? Because his argument is not grounded in reason; rather, it is grounded solely in Judaic religion and tradition. His argument is merely one from (religious) authority; there is literally no critical analysis of Jewish religious thinkers. In fact, it is exactly this lack of critical thinking that leads Rashi to condone the death penalty for certain crimes, while arguing that even criminals sentenced to death deserve to be treated with respect and humanity. This position is obviously logically inconsistent: how can the violation of a right as fundamental as the right to life possibly co-exist with respect and humanity? This position also contradicts a near consensus in the ethics and legal community that the death penalty is absolutely morally wrong as it breaches the right to life, which again, is a fundamental human right (The Lancet 2023). The latter, however, is not an issue for Rashi, who explicitly prioritizes religious obligations over human rights.

If these three publications are “religious contributions,” they are not of the same kind: the first relies on religious texts to enrich the discussion and to make its argument more compelling both philosophically and pragmatically; the second merely relies on a scholar who is associated with a religion; and the third relies on religious texts and tradition without any critical outlook. This suggests that the term as it is being used by Jecker et al. is either too wide to mean anything or that the praise of these publications (or of the journal in which they were published) as religious contributions has been out of place.

## The Destruction of Worlds

These three publications, however, do have something in common. They all directly or indirectly, implicitly or explicitly, entail the destruction of a world. They entail epistemic injustice as their existence and the arguments contained in them mean that a certain population does not receive an equally fair chance to give testimony nor feel they have an equal space at the bioethics table. This population, of course, is Palestinians.

Let us start from the most obvious. Rashi (2024, 58) writes about the “serious prohibition against spilling blood” and announces that Jewish Ethics “affirms not just the rights that each individual is born with but also the obligation incumbent upon every human being to reach out to and save fellow humans in their hour of distress” (59). Jewish ethics, according to Rashi vis-à-vis Maimonides, grounds a “mutual responsibility to care for others wherever they are” (2024, 61) during epidemics. Rashi further interprets Jewish ethics to oblige Jews to care about the health of their fellow humans: “love for another is expressed by a series of deeds that in fact every person would wish would be done for him, and he is required to do them himself” (2024, 62). He specifically cites Leviticus 19:16, “Do not do anything that endangers your neighbour’s life,” and Leviticus 19:18, “Love your neighbour as yourself.”

Rashi’s article was submitted in October 2021. He of course could not have been aware of the flood of Palestinian blood that would be spilled in the Occupied Palestinian Territory following Hamas’ attack 2 years into the future (World Health Organization 2024). Nor could he have known about the treatment—of lack thereof—of Palestinian prisoners in the wake of the current war in Gaza (Lederman, Davidovitch, and Lederman 2025). Rashi may even be unaware of the harm and lack of care inflicted by Jews on their Palestinian neighbours in the past seventy years (see below) But, he surely knew that Israel was not fulfilling its legal, ethical, and—even according to Rashi—religious obligation to provide COVID-19 vaccinations to Palestinians during the pandemic (Dahdal, et al. 2021; Lederman et al. 2022; Lederman, Majadli, and Lederman 2023).

Further, Rashi is affiliated with the University of Ariel. Ariel is a settlement in the West Bank, part of the Occupied Palestinian Territory. The West Bank was not granted to Israel by the United Nations and as part of the 1949 armistice agreement with Jordan. Israel conquered it during the 1967 war and has been governing over it to varying degrees ever since. Because the West Bank was taken by force rather than agreement, Israeli settlements there have been consistently declared illegal by the International Court of Justice (International Court of Justice 2004a, 2024b).

Specifically, the presence of settlers in the West Bank, the applicability of one set of laws for Palestinians and one for Jews, and the frequent violence towards Palestinians by settlers and the Israeli military violate several fundamental human rights of Palestinians granted to them by the International Covenant on Civil and Political Rights and other international legal instruments, namely the right to life; the right to liberty and security of person; the right to liberty of movement and freedom to choose his residence; the right to stand as equal before the courts; and the right not to be subjected to arbitrary or unlawful interference with his privacy, family, home or correspondence, nor to unlawful attacks on his honour and reputation. The fact then, that settlements and settler institutions such as Ariel University exist and the policies and behaviour by both the Israeli military and settlers invariably entail the destruction of the Palestinian world. They necessarily entail, in other words, eliminating “other ways of knowing, being, and acting.”

Consider Komesaroff next, who clearly writes with his heart on his sleeve. His article was submitted 24 January 2024. By that time, Israel has killed 25,295 people in Gaza, 70 per cent of whom were women and children. It injured 63,000 people and displaced 1.7 million. It conducted 318 Health attacks and detained sixty-one Health workers. In these circumstances, Komersaroff’s call for reconciliation, or “refiguring relationships to open up a space for dialogue to create pathways to heal the ruptures” (Komesaroff 2024, 29) seems out of place if not outright disrespectful. Expecting Palestinians to just forget the war in Gaza and generally seventy years of occupation, dispossession, and oppression and forgive Israel without any transitional justice means the destruction of their world and eliminating their way of knowing, being, and acting. Nelson Mandela demanded justice prior to any reconciliation with the oppressive white regime (Beinart 2013). Palestinians are allowed, and indeed should do the same. Justice in this context will include cessation of all aggressive legal and military measures, granting Palestinians their basic rights including the right to return to their homes and to self-sovereignty, and financial reparations. Only then reconciliation will be warranted and possible.

Also of note is the thinkers Komesaroff chooses to rely on: Habermas, Arendt, Levinas, Husserl, Bakhtin, etc. Only two references are written by Arabic/Islamic authors. Sukarieh (2019) is provided to support the claim that “Palestine is a sophisticated secular society with a refined culture that highly values peace and a highly educated population, even if the nature of educational practice is not immune from criticism” (Komesaroff 2024, 36). This is ironic, because this is not at all the main thrust of this article, and in fact it provides no empirical support for Komesaroff’s claim about the Palestinian population. The reference does critique the education system, but not at all in the way Komesaroff seems to appreciate. Sukarieh interviews Munir Fasheh, a learning theorist based in Ramallah. For the majority of the interview, Fasheh laments the Western, intellectual colonization of Palestinians, even while using the Arab language. Such colonization progressively subdues the Palestinian mind and harms the Palestinian People’s inner immune system or resilience. As if he knew of Komesaroff’s choice of Western (and Jewish) references, Fasheh recounts his own colonized upbringing in the Western educational system: “My mind was filled with a colonial language that used Arabic letters but western ideology”(Sukarieh 2019, 188).

Fasheh argues that “What a culture needs in order to flourish is a space where people live in accordance with their ways, in free associations with each other” (Sukarieh 2019, 193). Yet, this is exactly what Israel sought to prevent Palestinians from achieving: “During the intifada, Israel closed schools, universities, as well as other institutions” (Sukarieh 2019, 199). Fortunately, this Israeli attempt to curb free spaces for Palestinians backfired, and small, grassroots community groups emerged as part of the Palestinian resistance. These groups, in fact, convinced Fasheh that “small groups formed by people with no authority inside or from outside, and with deep attentiveness to what is happening around, are the backbone of society; they form a main factor in the immune system at the personal and communal levels” (Sukarieh 2019, 198).

The other irony here, both for Jecker et al. and for Komesaroff, is that in the quote mentioned Komesaroff seems to praise the Palestinian society for being secular. This neither aligns with pluriversality, nor with the reference itself, where Fasheh actually claims that the traditional, religious worldview is less dogmatic than the modern, Western, scientific worldview: “Whereas most people feel religions are dogmatic and science is much more open, I believe the opposite to be true” (Sukarieh 2019, 190). If anything, Jecker et al., as well as other Western readers, would have appreciated hearing about Fasheh’s discussion of the importance of humility and the term *Muthanna* in Arabic*,* or the relation between two people that becomes very important in their lives. Fasheh understands the logic of *Muthanna* to be expressed as “YOU are, therefore I am,” and argues that there could be no pluralistic co-habitation without it: “… without muthanna, it is difficult to develop a pluralistic attitude in living and perceiving” (Sukarieh 2019, 192). Fasheh even explicitly praises pluriversality: “Deepening the ‘pluriverse’ where various worlds enrich and nurture one another should be what we strive for as a vision” (Sukarieh 2019, 193).

All of this rich history and thought, unfortunately, is made to disappear by Komesaroff’s writing hands, and is instead reduced to the reference being misused. The reader, as a result, misses an opportunity to learn more Palestinian history and culture, Palestinians’ oppressions by Israel, and their resilience throughout the years.

Komesaroff’s use of the second Arabic/ Muslim reference is even worse. Komesaroff imagines a moral, political, and legal symmetry between Israel and Palestinians, arguing that real reconciliation will require grassroot commitments from both individuals and communities. He then cites Falah (2021) to support the suggestion that “the failure of the Oslo Accords, which were signed in 1993 and ultimately collapsed in 2002 following vigorous and implacable opposition from within both Palestinian and Jewish populations, provides proof of this” (Komesaroff 2024, 32). Again, this is not at all what Falah argues. Falah first laments the false symmetry often depicted by commentators who treat Israel and Palestinians as morally, legally, and politically equivalent. In fact, they are not: Israel is the occupier and has consistently enjoyed American support, while Palestinians have been occupied. Double speech and manipulations have often been employed by Israel to sway Palestinian and International opinion while in fact ignoring the requests and interests of Palestinians. Falah, then, makes a compelling case showing that Israel, while “talking the talk” of peace, never “walked the walk”–—the topic of analysis is the actions and policies of Israeli governments throughout the years, not the Israeli populations nor the Palestinian side at all:

Arguably, the main obstacles for moving forward with such an option was due to the fact that successive Israeli governments since 1993 were ostensibly advancing discursive talk, saying they seek a solution to the conflict based on the two-state formula—but in effect creating irreversible facts on the ground … with land acquisition and extensive Israeli settlement enclavization, aiming at preventing a viable Palestinian state to ever emerge in the Occupied Territories of June 1967. (Falah 2021)

Komesaroff’s article, then, is not an instance of openness, humility, and respect. Whether intentional or not, by his choice and (mis)use of references as well by advocating reconciliation without justice Komesaroff falsely creates a symmetry between an occupier and an occupied, and further contributes to the destruction of the world of Palestinians.

As to Lederman and Halberthal, one has to know the present as well as the historical context to appreciate the world that is being destroyed. While writing the article the first author was residing in Haifa. Often would he dine at his favourite hummus place in Wadi Nisnas, enjoying his financial, social, and political freedoms. Based on pictures, it is very much the same Wadi Nisnas that only seventy years ago was one of the six Arab neighbourhoods in Haifa, which prior to 1948 was home to sixty-five thousand Palestinians and seventy thousand Jews. It is the same Wadi Nisnas that saw daily fighting between Israeli militia groups and Palestinians between November 1947 and March 1948, with both sides committing terrorist acts, including the use of car bombs and mortar shells by the much more effective Israeli forces. This is how Benny Morris, a conservative Israeli historian, recounts one such act:

On the night of 4–5 March, a Haganah [predecessor of the current-day Israeli military] unit raided Wadi Nisnas with orders to “kill adult males.” They penetrated several houses, destroyed the furniture with Molotov Cocktails, and hit about 30 men, “among them 19 sure kills.” (Morris 2004, 106)

The Israeli attacks resulted in thirty thousand Palestinians fleeing their homes. During the Battle of Haifa, or Operation Bi’ur Hametz in April 21–22, 1948, fifteen thousand more Palestinians were forcefully expelled by Israel. As one historian puts it:

… the mass exodus of Haifa’s Arab population, which began on 22 April, was the spontaneous reaction to the ruthless combination of terror and psychological warfare tactics adopted by the Haganah during the attack. (Khalidi 2008, 30)

Such ruthless terror manifested, for instance, in the indiscriminate killing of Palestinian women and children by Israeli forces, as reported by a British intelligence officer:

During the morning [the Jews], were continually shooting down on all Arabs who moved both in Wadi Nisnas and the Old City. This included completely indiscriminate and revolting machinegun fire, mortar fire and sniping on women and children sheltering in churches and attempting to get out. (Morris 2004, 191)

By May 1948, only four to five thousand Palestinians remained in Haifa, and almost all were displaced to Wadi Nisnas which for all purposes had become a Palestinian ghetto. One reason for their displacement is that Jewish immigrants needed their homes (Morris 2004).

Historically speaking, then, the fact that a Jewish state exists in Palestine means that Palestinians there were denied safe existence as equals. The fact that the first author could live freely in Haifa and dine in Wadi Nisnas means that the same was denied of Palestinians. The hummus that he ate there is stained with Palestinian blood.

## Concluding Remarks

Nothing in what is said here denies the possibility of pluriversality in principle. The aim of this critique is rather to highlight one context in which any talk or praise of pluriversality is at best uncareful or worse outright malevolent. Komesaroff completely ignores another passage by Falah, linking Judaism, Zionism, colonization, and occupation:

Jewishness, Zionism, colonial aspirations of the Israeli political class “have been the dominant factors standing rigorously behind Israel’s performance strategy of denying Palestinians claims for self-determination and opting to continue its occupation of their lands, openly distancing itself from their commitment to abide by the Oslo Accords.” (Falah 2021)

In Israel, that link has been proven again and again to be unbreakable. Whenever religious rights take priority over human rights, there can be no pluriversality. Whenever justice is put aside for expediency, there can be no respect. And whenever ethicists do not take seriously their obligation to reproach others who do not share our world, there can only be a destruction of worlds.

## Data Availability

Not applicable.
